# Artificial Intelligence, Machine Learning, and Big Data for Ebola Virus Drug Discovery

**DOI:** 10.3390/ph16030332

**Published:** 2023-02-21

**Authors:** Samuel K. Kwofie, Joseph Adams, Emmanuel Broni, Kweku S. Enninful, Clement Agoni, Mahmoud E. S. Soliman, Michael D. Wilson

**Affiliations:** 1 Department of Biomedical Engineering, School of Engineering Sciences, College of Basic and Applied Sciences, University of Ghana, Accra P.O. Box LG 77, Ghana; 2 West African Centre for Cell Biology of Infectious Pathogens, Department of Biochemistry, Cell and Molecular Biology, University of Ghana, Accra P.O. Box LG 54, Ghana; 3 Department of Parasitology, Noguchi Memorial Institute for Medical Research, University of Ghana, Accra P.O. Box LG 581, Ghana; 4 Department of Medicine, Loyola University Medical Center, Maywood, IL 60153, USA; 5 Department of Biomedical Engineering, McKelvey School of Engineering, Washington University in St. Louis, St. Louis, MO 63105, USA; 6 Discipline of Pharmaceutical Sciences, School of Health Sciences, University of KwaZulu-Natal, Westville Campus, Durban 4001, South Africa; 7 Conway Institute of Biomolecular and Biomedical Research, School of Medicine, University College of Dublin, D04 V1W8 Dublin 4, Ireland

**Keywords:** drug discovery, deep learning, artificial intelligence, big data, Ebola virus, classifiers, machine learning

## Abstract

The effect of Ebola virus disease (EVD) is fatal and devastating, necessitating several efforts to identify potent biotherapeutic molecules. This review seeks to provide perspectives on complementing existing work on Ebola virus (EBOV) by discussing the role of machine learning (ML) techniques in the prediction of small molecule inhibitors of EBOV. Different ML algorithms have been used to predict anti-EBOV compounds, including Bayesian, support vector machine, and random forest algorithms, which present strong models with credible outcomes. The use of deep learning models for predicting anti-EBOV molecules is underutilized; therefore, we discuss how such models could be leveraged to develop fast, efficient, robust, and novel algorithms to aid in the discovery of anti-EBOV drugs. We further discuss the deep neural network as a plausible ML algorithm for predicting anti-EBOV compounds. We also summarize the plethora of data sources necessary for ML predictions in the form of systematic and comprehensive high-dimensional data. With ongoing efforts to eradicate EVD, the application of artificial intelligence-based ML to EBOV drug discovery research can promote data-driven decision making and may help to reduce the high attrition rates of compounds in the drug development pipeline.

## 1. Introduction

Since the emergence of Ebola virus disease (EVD), concerted efforts have been employed in the quest to identify inhibitors as potential biotherapeutic molecules. EVD is a deadly zoonotic disease caused by the Ebola virus from the filoviridae family [1]. Although the exact source of EBOV remains unknown, it believed to be animal-borne and associated with monkeys, chimpanzees, and apes, including humans [2]. EBOV is transmitted from human to human via direct contact or contact with the body fluid of an infected person [3]. A person infected with EVD shows symptoms of fever, aches, and pains such as severe headache; gastrointestinal symptoms, including diarrhea and vomiting; and multiple organ dysfunction syndromes [4]. The Ebola virus (EBOV) is an enveloped, single-stranded, negative-sense RNA virus [5]. It forms a threadlike shape, with a uniform diameter of 80 nm and a typical length between 970 nm and 1200 nm [6]. The genome of EBOV encodes seven structural proteins comprising nucleoprotein (NP), polymerase cofactor (VP35), Virion matrix protein (VP40), Glycoprotein, transcription activator (VP30), VP24, and RNA-dependent RNA-polymerase (L) [7]. 

A total of 33,604 Ebola virus infections in humans were recorded, with an average death rate of 43.8%, constituting more than 14,000 deaths, during the 2014–2016 outbreak in Guinea, Liberia, and Sierra Leone, as well as the 2018–2020 outbreak in Congo [3]. There is a re-emergence of the outbreak in Guinea and the Democratic Republic of Congo [8]. The highly virulent and lethal nature of EVD highlights the need to develop therapeutic agents that could limit the spread of the virus. This had led to several efforts to prioritize compounds in the search for small molecule inhibitors of the Ebola virus [9].

Currently, there are two Food and Drug Administration (FDA)-approved treatments for the Ebola virus. The first, Inmazeb, is a mixture of three monoclonal antibodies that targets the glycoprotein of the Ebola virus and blocks attachment and entry of the virus [10]. The second is a human monoclonal antibody, Ebanga, which blocks the binding of the virus to the cell receptor [11]. Nonetheless, several EVD drug discovery studies are underway. 

The advancement in large-scale biological experiments and data collection initiatives has spurred in silico studies geared towards the identification of small molecule inhibitors of disease targets [12]. Big data describes a voluminous amount of data that can be analyzed computationally to unravel trends and patterns [13,14]. Big data-driven drug development was employed in drug repurposing of FDA-approved drugs against other disease targets [12,15,16]. Other advanced applications of big data include the integration of gene expression analysis, cellular screening systems, and healthcare informatics to identify chemical structures of therapeutic relevance [17]. 

With the growth of chemical data from high-throughput screening for a target of interest, the search for a small molecule to interact with these targets is essential. Artificial intelligence (AI) and machine learning approaches provide a faster alternative to categorize compounds that possess therapeutic indications [18,19,20]. Big data has therefore heavily influenced the recent drug discovery paradigm. This article discusses perspectives on the role that AI plays in the identification of small molecule inhibitors and ML approaches utilized in the search for small molecule inhibitors of EVD. Furthermore, it highlights large-scale databases containing multitudes of bioactive compounds that could be utilized to train machine learning models. It also shows how novel ML techniques such as deep neural networks could be leveraged to develop efficient and robust models to prioritize compounds with high propensity to possess anti-Ebola virus activity.

Drug discovery and development are time-consuming and expensive, with numerous compounds failing at various stages during clinical trials [21,22]. AI and ML approaches applied to various stages of the drug development pipeline provide data-driven decision making and can reduce the timeline for drug development via predictions [18,23]. These predictions are faster and cheaper than wet laboratory experiments, which are laborious. AI approaches in drug discovery result in generating potential lead-like ligands for drug targets, and when predictions are based on suitable data, the likelihood of potent therapeutic compounds going into clinical trial stages increases [24]. ML is any technique that makes computers learn from previous data observations and improve their behavior for a given task [25]. ML models change their inputs into meaningful outputs, a process that is learned from exposure to known examples of input and output data. ML is categorized into three, namely supervised, unsupervised, and reinforcement learning [26]. Numerous ML algorithms have been exploited in the paradigm of drug discovery. The use of ML in drug discovery addresses the issue of drug candidate identification via the prediction of drug–target interactions [27]. More so, molecules emanating from de novo design generate chemical structures with desirable characteristics, which are leveraged computationally for the synthesis of novel molecules [28]. 

## 2. Machine Learning Algorithms Deployed in Ebola Virus Drug Discovery

ML algorithms are key determinants of the effectiveness and efficiency of ML in drug discovery. As such, employing an appropriate learning algorithm that is suitable for the respective application is prudent. Notable algorithms include artificial neural networks (ANN) [29,30], decision trees (DT) [31,32], support vector machines (SVM) [33], and cluster analysis (CA) [34,35,36]. 

Several reports have thus employed some of these algorithms in EBOV drug discovery. One of such earlier reports was a study by Erkins et al. in 2015, in which two different anti-EBOV-predicting Bayesian ML models were trained [9] on two groups of datasets comprising the viral pseudotype entry and the EBOV replication assays, with both constituting 868 compounds [37]. The trained models were then used to screen the MicroSource (http://www.msdiscovery.com/spectrum.html) (accessed on 26 January 2023) library of drugs to predict potential anti-EBOV compounds. For each of the training assay data, molecules with IC_50_ values < 50 µM were classified as actives and all others were considered inactive. The half-maximal inhibitory concentration (IC_50_) is a quantitative measure that shows how much of a particular inhibitory substance is required to inhibit a particular biological process by half, thus providing a measure of the drug’s efficacy [38]. The Bayesian model was trained on this dataset together with two other ML algorithms comprising SVM and Recursive Partitioning Forest using a 5-fold cross-validation technique. The Bayesian model performed the best, with a receiver operating characteristic (ROC) value of 0.86. The MicroSource library with 2320 drugs was scored with the trained Bayesian model, predicting tilorone, quinacrine, and pyronaridine tetraphosphate as potential anti-EBOV drugs. Tilorone, quinacrine, and pyronaridine tetraphosphate which had the highest scores from the ML predictions were experimentally validated in vitro and found to possess EC_50_ values of 350, 420, and 230 nM, respectively. These EC_50_ values were much lower than the positive control, chloroquine, with EC_50_ value of 4.0 µM. [9]. EC_50_ is a measure of the pharmacological effectiveness of a compound. It is the concentration of a compound at which the biological response is half of the maximum response [39]. The three compounds with lower EC_50_ values are able to achieve the same biological effect at lower concentration, indicating that they are more effective.

Moreover, the Bayesian approach employed in the prediction of activity spectra of substances [40] can be used to predict the antiviral activity of compounds to facilitate the identification of potentially bioactive molecules against the EBOV protein VP24 [41,42]. 

So far, the Bayesian, SVM, and recursive partitioning forest are the available algorithms that have been used to develop models to predict potential anti-EBOV compounds. Other machine learning algorithms, including single-layer artificial neural networks, decision trees, and logistic regression, have also been applied to other fields of EVD other than predicting biotherapeutic compounds [43,44]. For instance, an ensemble of the aforementioned algorithms was used to predict the disease prognosis and outcomes of EBOV patients with appreciable levels of performance [44]. 

A more recent application of ML in EBOV drug discovery is the work by Rajput and Kumar (2022) [45] in which SVM, random forest, and artificial neural networks were employed using tenfold cross-validation (Table 1). In their report, the best predictive model showed a Pearson’s correlation coefficient ranging from 0.83 to 0.98 on training/testing (T274) dataset. These models were subsequently cross-validated for robustness using William’s plot, following which the models were integrated into a web server. Another such recent application of ML in EBOV drug discovery is a report from our group (Table 1) where predictive models developed using five algorithms comprising random forest (RF), SVM, naïve Bayes (NB), k-nearest neighbor (kNN), and logistic regression were used to predict potential anti-Ebola virus small molecule inhibitors of EBOV glycoprotein and VP40 [46]. Our study employed EBOV cell entry inhibitors from the PubChem database as training data, where RF, LR, and SVM models also showed plausible performances with overall accuracy values of 0.89, 0.84, and 0.86, respectively. These three models were implemented as a web server known as “EBOLApred” which assigns a confidence score to the predicted bioactivity validated using the applicability domain concept [46]. Collectively, these reports highlight the crucial role of ML in EBOV drug discovery, leaving room for further exploration.

## 3. Limitations on the Use of Conventional ML Models to Predict Anti-EBOV Compounds

Conventional ML algorithms have certain drawbacks when used to predict Ebola virus inhibitors. One significant issue is the high dimensional data required to predict inhibitors. The complexity of the data needed to predict inhibitors poses a challenge for most conventional ML models to manage [47]. Another limitation is that predictive models may not be able to generalize well to new data. These models are often trained on a limited set of data and may not perform well when applied to new or unseen data [48]. This is particularly problematic when dealing with diseases such as Ebola, where the amount of data available may be limited. The risk of overfitting exists when using conventional ML techniques to predict Ebola virus inhibitors. Even while these models may perform well on training data, they have trouble generalizing to fresh data [49]. To overcome these limitations, researchers may need to turn to more advanced machine learning techniques such as deep learning, which have shown to be more effective in handling large and complex data [50]. Additionally, the use of more diverse and comprehensive data sets can also help to improve the accuracy of predictions. With the ongoing efforts to combat EVD, it is crucial to continue to develop and improve predictive models to aid in the identification of potential drug candidates and management of the disease.

## 4. Deep Neural Network as an Efficient and Robust Alternative to Predict Anti-EBOV Compounds

Deep learning has become the most dominant component of ML and an emerging technique for accelerating the prediction of small molecules of therapeutic relevance [51,52,53,54]. Deep learning algorithms are classic models in pattern recognition tasks, and due to their robustness, good performance, and simple form, they are widely used in solving nonlinear classification problems [55,56]. In a comparative study of various screening methods on the ChEMBL database as the benchmark, deep learning outperformed the other seven screening methods for the mean area under the curve (AUC) across 1230 drug targets [48]. The AUC of deep learning (0.830) surpassed the threshold of 0.8 for commercial models used for virtual screening of compounds, thus having the potential to become a standard method in drug design [52]. Another comparative study corroborated the efficiency of the deep neural network (DNN) over other ML methods, including support vector machines, random forests, naïve Bayes, and logistic regression models [57]. The superiority of DNN was further demonstrated in a report by Wallach et al. in 2015, in which AtomNet, a structure-based bioactivity prediction for small molecules, was built using a deep convolutional neural network and was shown to outperform Smina [58]. AtomNet was even shown to also predict active compounds for drug targets that have no known modulators [58,59]. In a separate report by Bilsland et al. in 2015, 3517 compounds were used to train a neural network in a machine learning-based virtual screening study, leading to the identification of potential senescence agonists [60]. The resulting classification model in the report was subsequently employed to screen about 2 million lead-like compounds, of which 147 hits were identified [61]. Among the 147 hits, CB-20903630, a benzimidazolone, demonstrated low micromolar IC_50_ via in vitro assays, and could be explored in the development of selective cell cycle inhibitors [60]. In 2020, Rifaioglu et al. [61] also employed a deep convolutional neural network to develop DEEPScreen, a drug–target interaction (DTI) predictive model. DEEPScreen predicted JAK proteins as new targets of the drug cladribine, which were confirmed experimentally [61]. Altogether, these reports highlight robust performance of deep learning; as such, its role in current-day drug design cannot be overemphasized.

Deep learning models have been used to predict inhibitors of drug targets [62,63]. With the emergence of severe acute respiratory syndrome coronavirus 2 (SARS-CoV2), the search for highly efficacious compounds to inhibit the activities of the virus has become more urgent and needful [64,65,66]. Among such reports is a study by Ton et al., in which Deep Docking (DD), a novel deep learning platform for structure-based virtual screening (SBVS) was developed and applied to screen 1.3 billion compounds to identify 1000 potential compounds for the SAR-CoV-2 main protease (Mpro) [67]. Though these predicted compounds need experimental validation, they serve as an essential prioritized list for further development. In another report, HIV-1 sequence data and drug resistance assays for 18 antiretroviral therapy (ART) drugs were used to develop three deep learning architectures to predict drug resistance [68]. The report identified a convolutional neural network as the best architecture when compared to multilayer perceptron and bidirectional recurrent neural networks [68]. A report by Yao et al. in 2020 and Wang et al. in 2021 also employed an artificial neural network (ANN) to develop an ontology-based model for predicting the side-effect of compounds, which was employed to evaluate the traditional Chinese medicine (TCM) prescriptions officially recommended for the treatment of coronavirus disease 2019 (COVID-19) [55,69]. 

Apart from antiviral compound prediction, deep learning has also been employed in other drug discovery efforts. One of such efforts was a study by Zhavoronkov et al. in 2019 [66], in which a DNN-based generative reinforcement learning model was developed to predict Discoidin domain receptor 1 (DDR 1) inhibitors. Two of the predicted compounds from the model strongly inhibited DDR1 activity in vitro, with IC_50_ of 10 and 21 nM [70]. In a recent report, Bhagwati et al. in developed a DNN model from the ChEMBL datasets to virtually screen the Mabridge database (https://www.maybridge.com) (accessed on 26 January 2023) [71]. The model from their report identified potential inhibitory molecules of renin protein, a protein involved in the development of hypertension and other cardiovascular diseases. In total, 8701 compounds were used for the training, of which 2628 compounds were classified as active. After validation, the DNN model had an accuracy of 99.83% and a Matthew correlation coefficient (MCC) value of 0.975, which represents a strong correlation between the actual and the predicted classification [71].

Deep learning algorithms, despite their outstanding performance in comparison to several machine learning algorithms in the prediction of small molecules or inhibitors of drug targets [72], are yet to be implemented in the quest to identify anti-EBOV compounds. DNN learns meaningful representations from data through successive layers. The layered representations are learned through models called neural networks, structured in layers stacked on top of each other. DNN can be viewed as a model that maps input to targets via a deep sequence of data transformations that are learned by exposure to examples [56]. It is therefore worth utilizing DNN models to predict anti-EBOV compounds since they have been shown to produce better performance. 

Although deep learning models are effective techniques to predict Ebola virus inhibitors, they do have some shortcomings that must be taken into account. The challenge of hyperparameter tuning is one of the key difficulties [73]. Before training the model, hyperparameters, which are parameters that govern the model’s behavior, are defined. The learning rate, the number of hidden layers, and the number of neurons in each layer are a few examples [74]. It can be difficult to optimize these hyperparameters, since there are frequently a large number of alternative combinations to explore, and finding the ideal combination might take a lot of computing effort [74]. Another drawback of using deep learning models for predicting Ebola virus inhibitors is the lack of computational tools for reproducibility. Deep learning models are frequently complicated and tricky to comprehend, and it might be difficult to replicate the outcomes of a certain study or experiment [75]. Due to this, it may be challenging to confirm a research’s findings or corroborate a similar study in another environment. This lack of reproducibility can pose a significant challenge when predicting inhibitors, where accurate and reproducible results are crucial. Furthermore, deep learning models are also computationally expensive; they require a significant amount of computational power and time to train [76]. This can be a significant obstacle, especially for researchers working in resource-constrained environments. Lastly, selecting the right ML model out of other predictive models can be a difficult task, as the performance of a model can vary depending on the specific task and dataset. Different models may perform well on different metrics, such as accuracy, precision, recall, F1 score, and AUC-ROC [77]. Some models may perform well on a specific subset of the data but poorly on the rest of it, which highlights the importance of considering the distribution and characteristics of the data. Furthermore, there may be trade-offs between different metrics, such as a model with high accuracy but low recall, and it is important to consider the specific requirements and goals of the task when selecting a model [77]. Overall, selecting the right ML model requires a thorough understanding of the data, the task, and the specific metrics that are relevant for that task.

## 5. Data Sources for Ebola Machine Learning Studies

**Figure 1:**
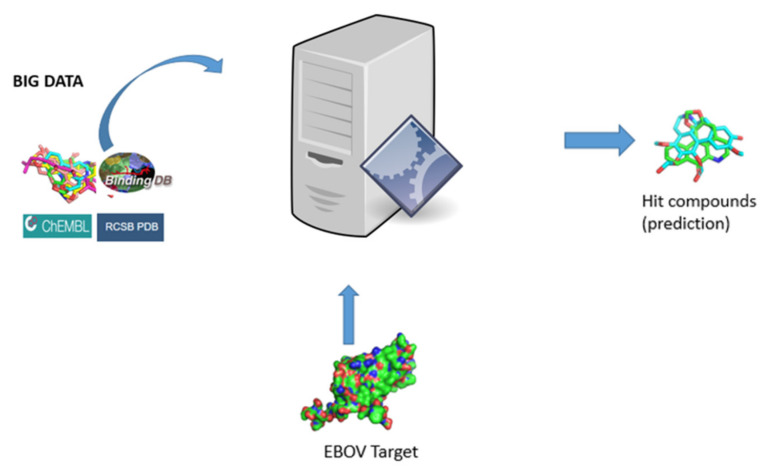
Schematic highlighting the integration of available resources and machine learning models (ML) to aid in potential anti-EBOV molecule prediction.

Finding anomalies or patterns and correlations within a large dataset to predict outcomes is a relevant technique in drug discovery [78]. With the advancement in the studies of the composition, structure, and interactions of cellular molecules, much information about drugs and targets is generated. Molecules with their structural features and biological activity data are curated in various databases for use in ML [79]. Many databases are available with increasing numbers of data sizes due to the accessibility of high-throughput data from around the globe [80]. As data and statistics about drugs and targets are gathered, it sets the pace for drug discovery studies. There are a number of databases with bioassay data on EVD that could be exploited for machine learning projects. Notable amongst such databases are PubChem [81], ChEMBL [82], BindingDB [83], DrugRepV [84] and Ebolabase [85], as highlighted in Figure 1. A search for the Ebola virus on PubChem yielded 597 bioassay data, whereas ChEMBL yielded 47 bioassay data assays (Table 2) that can be exploited for ML-based EBOV drug discovery studies. A search for Ebola virus data via BindingDB produced only 2, while a total of 868 compounds in DrugRepV were experimentally validated for anti-Ebola activities. A more comprehensive Ebola–human–drug interaction database was also recently created by Muthaiyan et al. in 2021 [85], curating 270 human proteins that interacted with EBOV, a database that was exploited for training models. Altogether, these datasets could be pooled to train ML models towards predicting anti-EBOV compounds with a good degree of therapeutic potential. 

Though the application of ML algorithms tends to hasten the pace in the quest for biotherapeutic compounds, the absence of vast and diverse datasets to train and test models on is one of the primary research gaps in the application of machine learning and big data for Ebola medication development [72]. Despite continuing initiatives to gather and exchange data, larger and more varied datasets are still required in order to create reliable and accurate models [86]. This is crucial for creating ML models that generalize well to new data and can be used in a variety of situations and populations. There is a need for greater study on the best ways to create and test novel compounds in clinical settings as well as how to transfer the findings from ML models into new medicines. This entails creating strategies for discovering novel therapeutic targets, forecasting the effectiveness of various drugs against the virus, and enhancing the planning and evaluation of clinical trials for the treatment of Ebola [87]. Additional studies are required to create numerous online platforms and applications, which are easy to utilize to support high-throughput screening of drugs with more precision and effectiveness. Overall, the application of big data and ML techniques have the potential to hasten the identification and creation of novel Ebola therapeutics, but more work remains to fully fulfill this promise.

## 6. Conclusions

Machine-learning algorithms such as support vector machines, Bayesian models, and recurrent partitioning forest have been used to predict potential anti-EBOV molecules from FDA-approved compounds. The full clinical potential of drug repurposing for EBOV treatment is yet to be realized. The dearth of available alternative FDA-approved drugs for the treatment of EBOV is a global concern. ML techniques are cost-effective and time-efficient strategies in developing predictive models for drugs. It is therefore imperative to leverage the vast amount of knowledge related to ML and available EBOV bioassay data to identify EBOV therapeutics to augment existing efforts geared towards eradicating the disease. The increasing amount of biological and pharmacological data coupled with existing and new ML methods will certainly provide new perspectives into therapeutic mechanisms. Furthermore, with increasing biological data, model applicability will be enhanced as better data integration techniques will emerge. The deep neural network is robust, with better performance in antiviral prediction, and can be employed to predict small molecule inhibitors of Ebola virus disease.

## Figures and Tables

**Table 1:** Machine learning algorithms employed anti-EBOV research work with their varying performances. The models consisted of support vector machines (SVM), recursive partitioning forest (RPF), random forest models (RF), artificial neural network (ANN), naïve Bayes models (NB) and k-nearest neighbor models (kNN). Evaluation metrics included accuracy (ACC), Pearson correlation coefficient (PCC), mean absolute error (MAE), and root-mean-square error (RMSE).

Applications (URLs)	Model Used	ACC	PCC	MAE	RMSE
Anti-EBOV machine learning models [9] (http://molsync.com/ebola/) (accessed on 26 January 2023)	Bayesian model	0.82–0.86			
	RPF	0.75–0.85			
	SVM	0.73–0.76			
Anti-Ebola initiative [45] (https://bioinfo.imtech.res.in/manojk/antiebola) (accessed on 26 January 2023)	SVM		0.83	0.33	0.47
	RF		0.98	0.19	0.28
	ANN		0.95	0.23	0.29
EBOLApred [46] (http://197.255.126.13:8000/) (accessed on 26 January 2023)	RF	0.80			
	SVM	0.86			
	NB	0.65			
	kNN	0.80			

**Table 2:** Ebola virus-related bioassay data available in ChEMBL [72]. The table consists of numbers of compounds, the description of the assays, and IDs. Assays with number of compounds less than 100 were excluded.

ChEMBL ID	Description	No. of Compounds
CHEMBL3562085	PubChem BioAssay. qHTS Assay for Identifying Compounds that block Entry of Ebola Virus: Screen1, ratio channel. (Class of assay: confirmatory).	558
CHEMBL3562152	PubChem BioAssay. qHTS Assay for Identifying Compounds that block Entry of Ebola Virus: Screen2, ratio channel. (Class of assay: confirmatory).	1822
CHEMBL3562088	PubChem BioAssay. qHTS Assay for Identifying Compounds that block Entry of Ebola Virus: Screen1, green channel. (Class of assay: confirmatory).	570
CHEMBL3562112	PubChem BioAssay. qHTS Assay for Identifying Compounds that block Entry of Ebola Virus: Screen1, blue channel. (Class of assay: confirmatory).	556
CHEMBL3562133	PubChem BioAssay. qHTS Assay for Identifying Compounds that block Entry of Ebola Virus, Screen1 green channel. (Class of assay: confirmatory).	570
CHEMBL3561981	PubChem BioAssay. qHTS Assay for Identifying Compounds that block Entry of Ebola Virus: Screen 4, blue channel. (Class of assay: confirmatory).	168
CHEMBL3562144	PubChem BioAssay. qHTS Assay for Identifying Compounds that block Entry of Ebola Virus: Screen 4, green channel. (Class of assay: confirmatory).	170
CHEMBL3562135	PubChem BioAssay. qHTS Assay for Identifying Compounds that block Entry of Ebola Virus, Screen 1 blue channel. (Class of assay: confirmatory).	556
CHEMBL3562064	PubChem BioAssay. qHTS Assay for Identifying Compounds that block Entry of Ebola Virus: Screen 2, blue channel. (Class of assay: confirmatory).	1996
CHEMBL3562033	PubChem BioAssay. qHTS Assay for Identifying Compounds that block Entry of Ebola Virus: Screen 4, ratio channel. (Class of assay: confirmatory)	170
CHEMBL3562136	PubChem BioAssay. qHTS Assay for Identifying Compounds that block Entry of Ebola Virus, Screen 2 blue channel. (Class of assay: confirmatory).	1968
CHEMBL3562119	PubChem BioAssay. qHTS Assay for Identifying Compounds that block Entry of Ebola Virus, Screen 2 green channel. (Class of assay: confirmatory).	2170
CHEMBL3561990	PubChem BioAssay. qHTS Assay for Identifying Compounds that block Entry of Ebola Virus: Screen 2, green channel. (Class of assay: confirmatory).	2189
CHEMBL3562146	PubChem BioAssay. qHTS Assay for Identifying Compounds that block Entry of Ebola Virus, Screen 2 ratio channel. (Class of assay: confirmatory).	1812
CHEMBL3561973	PubChem BioAssay. qHTS Assay for Identifying Compounds that block Entry of Ebola Virus, Screen 1 ratio channel. (Class of assay: confirmatory).	558

## Data Availability

Data sharing not applicable.

## References

[1] Emanuel J., Marzi A., Feldmann H. (2018). Filoviruses: Ecology, Molecular Biology, and Evolution. Adv. Virus Res..

[2] Sivanandy P., Jun P.H., Man L.W., Wei N.S., Mun N.F.K., Yii C.A.J., Ying C.C.X. (2022). A systematic review of Ebola virus disease outbreaks and an analysis of the efficacy and safety of newer drugs approved for the treatment of Ebola virus disease by the US Food and Drug Administration from 2016 to 2020. J. Infect. Public Health.

[3] Jacob S.T., Crozier I., Fischer W.A., Hewlett A., Kraft C.S., de La Vega M.-A., Soka M.J., Wahl V., Griffiths A., Bollinger L. (2020). Ebola virus disease. Nat. Rev. Dis. Prim..

[4] Rajak H., Jain D.K., Singh A., Sharma A.K., Dixit A. (2015). Ebola virus disease: Past, present and future. Asian Pac. J. Trop. Biomed..

[5] Farman A., Badshah S.L. (2020). Ebola, the Negative Stranded RNA Virus. Some RNA Viruses.

[6] Wan W., Kolesnikova L., Clarke M., Koehler A., Noda T., Becker S., Briggs J.A.G. (2017). Structure and assembly of the Ebola virus nucleocapsid. Nature.

[7] Qureshi A.I. (2016). Ebola Virus Disease: From Origin to Outbreak.

[8] World Health Organization Ebola Virus Disease. https://www.who.int/news-room/fact-sheets/detail/ebola-virus-disease.

[9] Ekins S., Freundlich J.S., Clark A.M., Anantpadma M., Davey R.A., Madrid P. (2015). Machine learning models identify molecules active against the Ebola virus in vitro. F1000Research.

[10] Markham A. (2021). REGN-EB3: First Approval. Drugs.

[11] Lee A. (2021). Ansuvimab: First Approval. Drugs.

[12] Qian T., Zhu S., Hoshida Y. (2019). Use of big data in drug development for precision medicine: An update. Expert Rev. Precis. Med. Drug. Dev..

[13] Brown N., Cambruzzi J., Cox P.J., Davies M., Dunbar J., Plumbley D., Sellwood M.A., Sim A., Williams-Jones B.I., Zwierzyna M. (2018). Big Data in Drug Discovery. Prog. Med. Chem..

[14] Mallappallil M., Sabu J., Gruessner A., Salifu M. (2020). A review of big data and medical research. SAGE Open. Med..

[15] Glicksberg B.S., Li L., Chen R., Dudley J., Chen B. (2019). Leveraging Big Data to Transform Drug Discovery. Methods Mol. Biol..

[16] Zhu H. (2020). Big Data and Artificial Intelligence Modeling for Drug Discovery. Annu. Rev. Pharmacol. Toxicol..

[17] Cha Y., Erez T., Reynolds I.J., Kumar D., Ross J., Koytiger G., Kusko R., Zeskind B., Risso S., Kagan E. (2018). Drug repurposing from the perspective of pharmaceutical companies. Br. J. Pharmacol..

[18] Dara S., Dhamercherla S., Jadav S.S., Babu C.H.M., Ahsan M.J. (2022). Machine Learning in Drug Discovery: A Review. Artif. Intell. Rev..

[19] Sarker I.H., Furhad M.H., Nowrozy R. (2021). AI-Driven Cybersecurity: An Overview, Security Intelligence Modeling and Research Directions. SN Comput. Sci..

[20] Priya S., Tripathi G., Singh D.B., Jain P., Kumar A. (2022). Machine learning approaches and their applications in drug discovery and design. Chem. Biol. Drug. Des..

[21] Xue H., Li J., Xie H., Wang Y. (2018). Review of Drug Repositioning Approaches and Resources. Int. J. Biol. Sci..

[22] Sun D., Gao W., Hu H., Zhou S. (2022). Why 90% of clinical drug development fails and how to improve it?. Acta Pharm. Sin. B.

[23] Vamathevan J., Clark D., Czodrowski P., Dunham I., Ferran E., Lee G., Li B., Madabhushi A., Shah P., Spitzer M. (2019). Applications of machine learning in drug discovery and development. Nat. Rev. Drug. Discov..

[24] Bender A., Cortés-Ciriano I. (2020). Artificial intelligence in drug discovery: What is realistic, what are illusions? Part 1: Ways to make an impact, and why we are not there yet. Drug. Discov. Today.

[25] Helm J.M., Swiergosz A.M., Haeberle H.S., Karnuta J.M., Schaffer J.L., Krebs V.E., Spitzer A.I., Ramkumar P.N. (2020). Machine Learning and Artificial Intelligence: Definitions, Applications, and Future Directions. Curr. Rev. Musculoskelet. Med..

[26] Nguyen G., Dlugolinsky S., Bobák M., Tran V., López García Á., Heredia I., Malík P., Hluchý L. (2019). Machine Learning and Deep Learning frameworks and libraries for large-scale data mining: A survey. Artif. Intell. Rev..

[27] Rifaioglu A.S., Atas H., Martin M.J., Cetin-Atalay R., Atalay V., Doğan T. (2019). Recent applications of deep learning and machine intelligence on in silico drug discovery: Methods, tools and databases. Brief. Bioinform..

[28] Schneider G., Clark D.E. (2019). Automated De Novo Drug Design: Are We Nearly There Yet?. Angew. Chem. Int. Ed..

[29] Deng J., Yang Z., Ojima I., Samaras D., Wang F. (2022). Artificial intelligence in drug discovery: Applications and techniques. Brief. Bioinform..

[30] Jiménez-Luna J., Grisoni F., Schneider G. (2020). Drug discovery with explainable artificial intelligence. Nat. Mach. Intell..

[31] Blower E.P., Cross P.K. (2006). Decision Tree Methods in Pharmaceutical Research. Curr. Top. Med. Chem..

[32] Schöning V., Hammann F. (2018). How far have decision tree models come for data mining in drug discovery?. Expert Opin. Drug. Discov..

[33] Rodríguez-Pérez R., Bajorath J. (2022). Evolution of Support Vector Machine and Regression Modeling in Chemoinformatics and Drug Discovery. J. Comput. Aided. Mol. Des..

[34] Ma J., Wang J., Ghoraie L.S., Men X., Haibe-Kains B., Dai P. (2019). A Comparative Study of Cluster Detection Algorithms in Protein–Protein Interaction for Drug Target Discovery and Drug Repurposing. Front. Pharmacol..

[35] Lund B., Ma J. (2021). A review of cluster analysis techniques and their uses in library and information science research: And clustering. Perform. Meas. Metr..

[36] Jaeger A., Banks D. (2022). Cluster analysis: A modern statistical review. WIREs Comput. Stat..

[37] Jiang X., Kopp-Schneider A. (2014). Summarizing EC50 estimates from multiple dose-response experiments: A comparison of a meta-analysis strategy to a mixed-effects model approach. Biom. J..

[38] Madrid P.B., Chopra S., Manger I.D., Gilfillan L., Keepers T.R., Shurtleff A.C., Green C.E., Iyer L.V., Dilks H.H., Davey R.A. (2013). A systematic screen of FDA-approved drugs for inhibitors of biological threat agents. PLoS ONE.

[39] Aykul S., Martinez-Hackert E. (2016). Determination of half-maximal inhibitory concentration using biosensor-based protein interaction analysis. Anal. Biochem..

[40] Parasuraman S. (2011). Prediction of activity spectra for substances. J. Pharmacol. Pharmacother..

[41] Kwofie S.K., Broni E., Teye J., Quansah E., Issah I., Wilson M.D., Miller W.A., Tiburu E.K., Bonney J.H.K. (2019). Pharmacoinformatics-based identification of potential bioactive compounds against Ebola virus protein VP24. Comput. Biol. Med..

[42] Darko L.K.S., Broni E., Amuzu D.S.Y., Wilson M.D., Parry C.S., Kwofie S.K. (2021). Computational Study on Potential Novel Anti-Ebola Virus Protein VP35 Natural Compounds. Biomedicines.

[43] Loubet P., Palich R., Kojan R., Peyrouset O., Danel C., Nicholas S., Conde M., Porten K., Augier A., Yazdanpanah Y. (2016). Development of a prediction model for ebola virus disease: A retrospective study in nzérékoré ebola treatment center, Guinea. Am. J. Trop. Med. Hyg..

[44] Colubri A., Silver T., Fradet T., Retzepi K., Fry B., Sabeti P. (2016). Transforming Clinical Data into Actionable Prognosis Models: Machine-Learning Framework and Field-Deployable App to Predict Outcome of Ebola Patients. PLoS Negl. Trop. Dis..

[45] Rajput A., Kumar M. (2022). Anti-Ebola: An initiative to predict Ebola virus inhibitors through machine learning. Mol. Divers..

[46] Adams J., Agyenkwa-Mawuli K., Agyapong O., Wilson M.D., Kwofie S.K. (2022). EBOLApred: A machine learning-based web application for predicting cell entry inhibitors of the Ebola virus. Comput. Biol. Chem..

[47] Bai Z., Krishnaiah P.R., Meyers R.A. (2003). Reduction of Dimensionality. Encyclopedia of Physical Science and Technology.

[48] Hussain J.N. (2020). High Dimensional Data Challenges in Estimating Multiple Linear Regression. J. Phys. Conf. Ser..

[49] Peng Y., Nagata M.H. (2020). An empirical overview of nonlinearity and overfitting in machine learning using COVID-19 data. Chaos Solitons Fractals.

[50] Wang H., Hu X. (2023). Deep Learning in Bioinformatics and Biomedicine. Methods.

[51] Carpenter K.A., Cohen D.S., Jarrell J.T., Huang X. (2018). Deep learning and virtual drug screening. Future Med. Chem..

[52] Unterthiner T., Mayr A., Klambauer G., Steijaert M., Wegner J., Ceulemans H., Hochreiter S. (2014). Deep learning as an opportunity in virtual screening. Adv. Neural Inf. Process. Syst..

[53] Goh G.B., Hodas N.O., Vishnu A. (2017). Deep learning for computational chemistry. J. Comput. Chem..

[54] Nag S., Baidya A.T.K., Mandal A., Mathew A.T., Das B., Devi B., Kumar R. (2022). Deep learning tools for advancing drug discovery and development. 3 Biotech.

[55] Wang Z., Li L., Yan J., Yao Y. (2020). Evaluating the Traditional Chinese Medicine (TCM) Officially Recommended in China for COVID-19 Using Ontology-Based Side-Effect Prediction Framework (OSPF) and Deep Learning. https://Www.Preprints.Org/Manuscript/202002.0230/V1.

[56] Chen H., Engkvist O., Wang Y., Olivecrona M., Blaschke T. (2018). The rise of deep learning in drug discovery. Drug. Discov. Today.

[57] Lenselink E.B., Ten Dijke N., Bongers B., Papadatos G., Van Vlijmen H.W.T., Kowalczyk W., Ijzerman A.P., Van Westen G.J.P. (2017). Beyond the hype: Deep neural networks outperform established methods using a ChEMBL bioactivity benchmark set. J. Cheminform..

[58] Wallach I., Dzamba M., Heifets A. (2015). AtomNet: A Deep Convolutional Neural Network for Bioactivity Prediction in Structure-based Drug Discovery. arXiv.

[59] Stecula A., Hussain M.S., Viola R.E. (2020). Discovery of novel inhibitors of a critical brain enzyme using a homology model and a deep convolutional neural network. J. Med. Chem..

[60] Bilsland A.E., Pugliese A., Liu Y., Revie J., Burns S., McCormick C., Cairney C.J., Bower J., Drysdale M., Narita M. (2015). Identification of a Selective G1-phase Benzimidazolone Inhibitor by a Senescence-Targeted Virtual Screen Using Artificial Neural Networks. Neoplasia.

[61] Rifaioglu A.S., Nalbat E., Atalay V., Martin M.J., Cetin-Atalay R., Doǧan T. (2020). DEEPScreen: High performance drug-target interaction prediction with convolutional neural networks using 2-D structural compound representations. Chem. Sci..

[62] Karki N., Verma N., Trozzi F., Tao P., Kraka E., Zoltowski B. (2021). Predicting Potential SARS-COV-2 Drugs-In Depth Drug Database Screening Using Deep Neural Network Framework SSnet, Classical Virtual Screening and Docking. Int. J. Mol. Sci..

[63] Zhang Y., Ye T., Xi H., Juhas M., Li J. (2021). Deep Learning Driven Drug Discovery: Tackling Severe Acute Respiratory Syndrome Coronavirus 2. Front. Microbiol..

[64] Zhang H., Saravanan K.M., Yang Y., Hossain M.T., Li J., Ren X., Pan Y., Wei Y. (2020). Deep Learning Based Drug Screening for Novel Coronavirus 2019-nCov. Interdiscip. Sci. Comput. Life Sci..

[65] Beck B.R., Shin B., Choi Y., Park S., Kang K. (2020). Predicting commercially available antiviral drugs that may act on the novel coronavirus (SARS-CoV-2) through a drug-target interaction deep learning model. Comput. Struct. Biotechnol. J..

[66] Bung N., Krishnan S.R., Bulusu G., Roy A. (2020). De novo design of new chemical entities (NCEs) for SARS-CoV-2 using artificial intelligence. ChemRxiv.

[67] Ton A.T., Gentile F., Hsing M., Ban F., Cherkasov A. (2020). Rapid Identification of Potential Inhibitors of SARS-CoV-2 Main Protease by Deep Docking of 1.3 Billion Compounds. Mol. Inform..

[68] Steiner M.C., Gibson K.M., Crandall K.A. (2020). Drug resistance prediction using deep learning techniques on HIV-1 sequence data. Viruses.

[69] Yao Y., Wang Z., Li L., Lu K., Liu R., Liu Z., Yan J. (2019). An Ontology-Based Artificial Intelligence Model for Medicine Side-Effect Prediction: Taking Traditional Chinese Medicine as an Example. Comput. Math. Methods Med..

[70] Zhavoronkov A., Ivanenkov Y.A., Aliper A., Veselov M.S., Aladinskiy V.A., Aladinskaya A.V., Terentiev V.A., Polykovskiy D.A., Kuznetsov M.D., Asadulaev A. (2019). Deep learning enables rapid identification of potent DDR1 kinase inhibitors. Nat. Biotechnol..

[71] Bhagwati S., Siddiqi M.I. (2022). Deep neural network modeling based virtual screening and prediction of potential inhibitors for renin protein. J. Biomol. Struct. Dyn..

[72] Wang M., Hou S., Wei Y., Li D., Lin J. (2021). Discovery of novel dual adenosine A1/A2A receptor antagonists using deep learning, pharmacophore modeling and molecular docking. PLoS Comput. Biol..

[73] Mahdaddi A., Meshoul S., Belguidoum M. (2021). EA-based hyperparameter optimization of hybrid deep learning models for effective drug-target interactions prediction. Expert Syst. Appl..

[74] Koutsoukas A., Monaghan K.J., Li X., Huan J. (2017). Deep-learning: Investigating deep neural networks hyper-parameters and comparison of performance to shallow methods for modeling bioactivity data. J. Cheminform..

[75] Isdahl R., Gundersen O.E. (2019). Out-of-the-Box Reproducibility: A Survey of Machine Learning Platforms.

[76] Thompson N.C., Greenewald K., Lee K., Manso G.F. (2020). The Computational Limits of Deep Learning. http://arxiv.org/abs/2007.05558.

[77] Dinga R., Penninx B.W.J.H., Veltman D.J., Schmaal L., Marquand A.F. (2019). Beyond accuracy: Measures for assessing machine learning models, pitfalls and guidelines. BioRxiv.

[78] Rodrigues T., Bernardes G.J.L. (2020). Machine learning for target discovery in drug development. Curr. Opin. Chem. Biol..

[79] Papadatos G., Gaulton A., Hersey A., Overington J.P. (2015). Activity, assay and target data curation and quality in the ChEMBL database. J. Comput. Aided. Mol. Des..

[80] Potemkin V., Potemkin A., Grishina M. (2018). Internet Resources for Drug Discovery and Design. Curr. Top. Med. Chem..

[81] Kim S., Thiessen P.A., Bolton E.E., Chen J., Fu G., Gindulyte A., Han L., He J., He S., Shoemaker B.A. (2016). PubChem substance and compound databases. Nucleic Acids Res..

[82] Gaulton A., Bellis L.J., Bento A.P., Chambers J., Davies M., Hersey A., Light Y., McGlinchey S., Michalovich D., Al-Lazikani B. (2012). ChEMBL: A large-scale bioactivity database for drug discovery. Nucleic Acids Res..

[83] Wassermann A.M., Bajorath J. (2011). BindingDB and ChEMBL: Online compound databases for drug discovery. Expert Opin. Drug. Discov..

[84] Rajput A., Kumar A., Megha K., Thakur A., Kumar M. (2021). DrugRepV: A compendium of repurposed drugs and chemicals targeting epidemic and pandemic viruses. Brief. Bioinform..

[85] Muthaiyan M., Naorem L.D., Seenappa V., Pushan S.S., Venkatesan A. (2021). Ebolabase: Zaire ebolavirus-human protein interaction database for drug-repurposing. Int. J. Biol. Macromol..

[86] Sarker I.H. (2021). Machine Learning: Algorithms, Real-World Applications and Research Directions. SN Comput. Sci..

[87] Jarada T.N., Rokne J.G., Alhajj R. (2020). A review of computational drug repositioning: Strategies, approaches, opportunities, challenges, and directions. J. Cheminform..

